# Tetrakis[(benzene-18-crown-6)potassium]bis[tris(thiocyanato)copper(I)]

**DOI:** 10.1107/S160053680800442X

**Published:** 2008-02-20

**Authors:** Xing-Min Song, Xian-Qiang Huang, Jian-Min Dou, Da-Cheng Li

**Affiliations:** a College of Chemistry and Chemical Engineering, Liaocheng University, Shandong 252059, People’s Republic of China

## Abstract

The title complex, bis­(μ-benzene-18-crown-6)-3κ^6^ *O*:4κ*O*;4κ^6^ *O*:3κ*O*-bis­(benzene-18-crown-6)-1κ^6^ *O*,6κ^6^ *O*-tetra-μ-thiocyanato-1:2κ^2^ *S*:*N*;2:3κ^2^ *N*:*S*;4:5κ^2^ *S*:*N*;5:6κ^2^ *N*:*S*-dithio­cyanato-2κ*N*,5κ*N*-2,5-dicopper(I)-1,3,4,6-tetra­potassium(I), [K_4_Cu_2_(NCS)_6_(C_16_H_24_O_6_)_4_] or {[K(C_16_H_24_O_6_)]_4_[Cu(NCS)_3_]_2_}, consists of four [K(benzene-18-crown-6)]^+^ cations and two [Cu(NCS)_3_]^2−^ anions, forming a dimeric structure with site symmetry 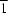. In each [Cu(NCS)_3_]^2−^ anion, the Cu^I^ atom is coordinated by three N atoms of thio­cyanate ligands in a trigonal–planar coordination geometry. Each [Cu(NCS)_3_]^2−^ anion bridges two [K(benzene-18-crown-6)]^+^ cations, with K—S distances of 3.317 (3) and 3.198 (3) Å, and two [K(benzene-18-crown-6)]^+^ cations are linked across a crystallographic centre of inversion, with K—O distances of 2.903 (5) Å.

## Related literature

For structures incorporating [Cu(NCS)_3_]^2−^ anions, see: Rusanova *et al.* (2000 ▶); Wang *et al.* (1987 ▶). For polymeric structures incorporating crown ethers, see: Desai *et al.* (2001 ▶); Bastos *et al.* (2000 ▶).
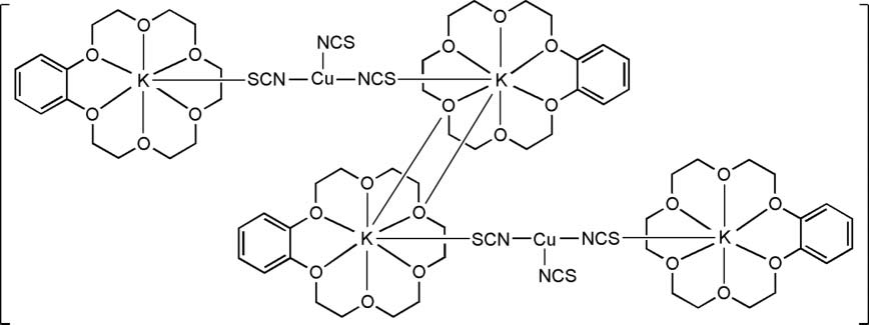


## Experimental

### 

#### Crystal data

[K_4_Cu_2_(NCS)_6_(C_16_H_24_O_6_)_4_]
*M* *_r_* = 1881.36
Triclinic, 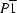

*a* = 9.702 (3) Å
*b* = 13.119 (4) Å
*c* = 17.968 (6) Å
α = 91.015 (6)°
β = 97.167 (6)°
γ = 105.558 (6)°
*V* = 2182.9 (12) Å^3^
*Z* = 1
Mo *K*α radiation
μ = 0.89 mm^−1^
*T* = 273 (2) K
0.49 × 0.21 × 0.16 mm

#### Data collection

Bruker SMART CCD diffractometer
Absorption correction: multi-scan (*SADABS*; Sheldrick, 1996 ▶) *T*_min_ = 0.668, *T*_max_ = 0.870
11606 measured reflections
7633 independent reflections
2441 reflections with *I* > 2σ(*I*)
*R*_int_ = 0.076

#### Refinement

*R*[*F* ^2^ > 2σ(*F* ^2^)] = 0.062
*wR*(*F* ^2^) = 0.160
*S* = 0.85
7633 reflections
505 parameters
H-atom parameters constrained
Δρ_max_ = 0.41 e Å^−3^
Δρ_min_ = −0.34 e Å^−3^

### 

Data collection: *SMART* (Bruker, 1997 ▶); cell refinement: *SAINT* (Bruker, 1997 ▶); data reduction: *SAINT*; program(s) used to solve structure: *SHELXS97* (Sheldrick, 2008 ▶); program(s) used to refine structure: *SHELXL97* (Sheldrick, 2008 ▶); molecular graphics: *SHELXTL* (Sheldrick, 2008 ▶); software used to prepare material for publication: *SHELXTL*.

## Supplementary Material

Crystal structure: contains datablocks I, global. DOI: 10.1107/S160053680800442X/bi2279sup1.cif

Structure factors: contains datablocks I. DOI: 10.1107/S160053680800442X/bi2279Isup2.hkl

Additional supplementary materials:  crystallographic information; 3D view; checkCIF report

## Figures and Tables

**Table:** 

Cu1—N1	1.873 (8)
Cu1—N2	1.910 (7)
Cu1—N3	1.915 (7)
K1—O5	2.683 (5)
K1—O2	2.718 (5)
K1—O1	2.727 (5)
K1—O3	2.756 (5)
K1—O6	2.761 (5)
K1—O4	2.875 (5)
K1—O4^i^	2.903 (5)
K1—S1	3.317 (3)
K2—O12	2.729 (5)
K2—O11	2.730 (5)
K2—O9	2.765 (5)
K2—O8	2.773 (5)
K2—O10	2.797 (5)
K2—O7	2.819 (5)
K2—S2	3.198 (3)

**Table:** 

N1—Cu1—N2	121.2 (3)
N1—Cu1—N3	121.5 (3)
N2—Cu1—N3	117.1 (3)

Symmetry code: (i) 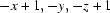.

**Fig. 1.**
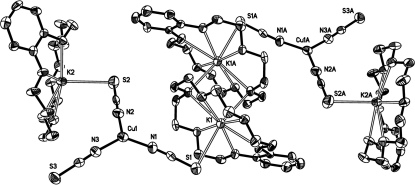
The title complex (H atoms omitted) showing the numbering scheme for Cu, K, N and S atoms. Displacement ellipsoids are drawn at the 30% probability level. Atoms with the suffix A are generated by the symmetry operator 1 - x, -y, 1 - z.

## supplementary crystallographic information

### Comment

Much interest has been focused on crown ethers and their metal cations, because they can act as modules to form polymeric supramolecular structures (Desai *et al.*, 2001; Bastos *et al.*, 2000).

The title complex consists of four [*K*(benzene-18-crown-6)]^+^ cations and two [Cu(NCS)_3_]^2-^ anions. The Cu^I^ atom is coordinated by three N atoms in a trigonal-planar geometry (Table 1). The K^+^ ions are coordinated by six O atoms, and lie approximately in the plane of the crown ether. The K—O bond lengths vary from 2.683 (5)–2.903 (5) Å for K1 and 2.729 (5)–2.819 (5) Å for K2. In addition, K^+^ is coordinated by one S atom from [Cu(NCS)_3_]^2-^, with K1—S1 = 3.317 (3) Å and K2—S2 = 3.198 (3) Å.

### Experimental

Cu(NCS) (0.1853 g, 1.5 mmol) and K(NCS) (0.1448 g, 1.5 mmol) in 15 ml absolute alcohol were refluxed for 4 h, then benzene-18-crown-6 (0.4675 g, 1.5 mmol) was added slowly and the mixture was refluxed for a further 4 h. After cooling to room temperature, the mixture was filtered and the solid recrystallized from diethyl ether. Elemental analysis: calculated C 44.69, H 5.14, N 4.47%; found: C 44.76, H 5.17, N 4.51%.

### Refinement

H atoms were positioned geometrically and refined using a riding model with C—H = 0.93–0.97 Å and *U*_iso_(H) = 1.2*U*_eq_(C).

### Crystal data

**Table:**

[K_4_Cu_2_(NCS)_6_(C_16_H_24_O_6_)_4_]	*Z* = 1
*M**_r_* = 1881.36	*F*_000_ = 980
Triclinic, *P*1	*D*_x_ = 1.431 Mg m^−^^3^
Hall symbol: -P 1	Mo *K*α radiation λ = 0.71073 Å
*a* = 9.702 (3) Å	Cell parameters from 1026 reflections
*b* = 13.119 (4) Å	θ = 2.3–19.0º
*c* = 17.968 (6) Å	µ = 0.89 mm^−^^1^
α = 91.015 (6)º	*T* = 273 (2) K
β = 97.167 (6)º	Block, green
γ = 105.558 (6)º	0.49 × 0.21 × 0.16 mm
*V* = 2182.9 (12) Å^3^	

### Data collection

**Table:**

Bruker SMART CCD diffractometer	7633 independent reflections
Radiation source: fine-focus sealed tube	2441 reflections with *I* > 2σ(*I*)
Monochromator: graphite	*R*_int_ = 0.077
*T* = 273(2) K	θ_max_ = 25.0º
ω scans	θ_min_ = 1.9º
Absorption correction: multi-scan(SADABS; Sheldrick, 1996)	*h* = −11→11
*T*_min_ = 0.669, *T*_max_ = 0.870	*k* = −11→15
11606 measured reflections	*l* = −21→19

### Refinement

**Table:**

Refinement on *F*^2^	Secondary atom site location: difference Fourier map
Least-squares matrix: full	Hydrogen site location: inferred from neighbouring sites
*R*[*F*^2^ > 2σ(*F*^2^)] = 0.062	H-atom parameters constrained
*wR*(*F*^2^) = 0.160	*w* = 1/[σ^2^(*F*_o_^2^) + (0.0464*P*)^2^] where *P* = (*F*_o_^2^ + 2*F*_c_^2^)/3
*S* = 0.85	(Δ/σ)_max_ < 0.001
7633 reflections	Δρ_max_ = 0.41 e Å^−^^3^
505 parameters	Δρ_min_ = −0.34 e Å^−^^3^
Primary atom site location: structure-invariant direct methods	Extinction correction: none

### Special details

**Table:**

Geometry. All e.s.d.'s (except the e.s.d. in the dihedral angle between two l.s. planes) are estimated using the full covariance matrix. The cell e.s.d.'s are taken into account individually in the estimation of e.s.d.'s in distances, angles and torsion angles; correlations between e.s.d.'s in cell parameters are only used when they are defined by crystal symmetry. An approximate (isotropic) treatment of cell e.s.d.'s is used for estimating e.s.d.'s involving l.s. planes.
Refinement. Refinement of *F*^2^ against ALL reflections. The weighted *R*-factor *wR* and goodness of fit *S* are based on *F*^2^, conventional *R*-factors *R* are based on *F*, with *F* set to zero for negative *F*^2^. The threshold expression of *F*^2^ > σ(*F*^2^) is used only for calculating *R*-factors(gt) *etc*. and is not relevant to the choice of reflections for refinement. *R*-factors based on *F*^2^ are statistically about twice as large as those based on *F*, and *R*- factors based on ALL data will be even larger.

### Fractional atomic coordinates and isotropic or equivalent isotropic displacement parameters (Å^2^)

**Table:**

	*x*	*y*	*z*	*U*_iso_*/*U*_eq_	
Cu1	−0.10846 (12)	−0.10295 (8)	0.25933 (6)	0.0631 (4)	
K1	0.35951 (18)	−0.15952 (12)	0.48793 (9)	0.0492 (5)	
K2	−0.03041 (19)	0.27283 (12)	0.09562 (10)	0.0566 (5)	
N1	−0.0459 (8)	−0.1929 (6)	0.3274 (4)	0.072 (2)	
N2	0.0190 (8)	0.0283 (5)	0.2376 (4)	0.066 (2)	
N3	−0.2976 (8)	−0.1432 (5)	0.2030 (4)	0.065 (2)	
O1	0.4114 (6)	−0.3250 (4)	0.5657 (3)	0.0529 (14)	
O2	0.4960 (5)	−0.2967 (4)	0.4361 (3)	0.0490 (14)	
O3	0.4511 (5)	−0.1319 (4)	0.3487 (3)	0.0501 (14)	
O4	0.3773 (5)	0.0397 (4)	0.4201 (3)	0.0502 (14)	
O5	0.1858 (5)	−0.0452 (4)	0.5271 (3)	0.0542 (14)	
O6	0.2402 (5)	−0.2078 (4)	0.6189 (3)	0.0523 (14)	
O7	−0.2852 (5)	0.2777 (4)	0.1540 (3)	0.0526 (15)	
O8	−0.0563 (6)	0.4250 (4)	0.1980 (3)	0.0598 (15)	
O9	0.1587 (6)	0.4733 (4)	0.1018 (3)	0.0632 (16)	
O10	0.1230 (6)	0.3411 (4)	−0.0244 (3)	0.0571 (15)	
O11	−0.0473 (6)	0.1308 (4)	−0.0203 (3)	0.0626 (16)	
O12	−0.2782 (6)	0.1135 (4)	0.0589 (3)	0.0632 (16)	
S1	0.0692 (3)	−0.34102 (19)	0.40627 (13)	0.0706 (7)	
S2	0.2182 (3)	0.21181 (19)	0.20496 (16)	0.0946 (9)	
S3	−0.5419 (3)	−0.21268 (18)	0.09955 (14)	0.0814 (8)	
C1	0.5322 (8)	−0.3583 (6)	0.5560 (5)	0.043 (2)	
C2	0.6081 (10)	−0.4018 (6)	0.6090 (5)	0.064 (2)	
H2	0.5798	−0.4109	0.6566	0.077*	
C3	0.7264 (10)	−0.4328 (7)	0.5929 (6)	0.075 (3)	
H3	0.7771	−0.4632	0.6294	0.090*	
C4	0.7697 (10)	−0.4188 (7)	0.5231 (6)	0.075 (3)	
H4	0.8489	−0.4403	0.5117	0.090*	
C5	0.6951 (9)	−0.3726 (6)	0.4703 (5)	0.058 (2)	
H5	0.7252	−0.3617	0.4232	0.069*	
C6	0.5774 (9)	−0.3425 (6)	0.4857 (5)	0.045 (2)	
C7	0.5258 (9)	−0.2889 (6)	0.3594 (4)	0.057 (2)	
H7A	0.5144	−0.3588	0.3367	0.069*	
H7B	0.6241	−0.2467	0.3577	0.069*	
C8	0.4219 (9)	−0.2380 (6)	0.3185 (4)	0.058 (2)	
H8A	0.4317	−0.2378	0.2654	0.070*	
H8B	0.3239	−0.2771	0.3240	0.070*	
C9	0.3642 (8)	−0.0726 (6)	0.3085 (4)	0.057 (2)	
H9A	0.2627	−0.1046	0.3116	0.069*	
H9B	0.3783	−0.0711	0.2560	0.069*	
C10	0.4111 (8)	0.0383 (6)	0.3445 (5)	0.062 (2)	
H10A	0.5144	0.0669	0.3450	0.075*	
H10B	0.3632	0.0832	0.3150	0.075*	
C11	0.2444 (8)	0.0633 (6)	0.4253 (5)	0.058 (2)	
H11A	0.1666	0.0118	0.3945	0.069*	
H11B	0.2482	0.1329	0.4068	0.069*	
C12	0.2161 (9)	0.0610 (6)	0.5048 (5)	0.062 (2)	
H12A	0.2999	0.1046	0.5368	0.074*	
H12B	0.1346	0.0890	0.5099	0.074*	
C13	0.1522 (8)	−0.0568 (6)	0.6012 (5)	0.062 (2)	
H13A	0.0718	−0.0280	0.6070	0.074*	
H13B	0.2346	−0.0182	0.6363	0.074*	
C14	0.1140 (8)	−0.1704 (6)	0.6171 (4)	0.061 (2)	
H14A	0.0774	−0.1800	0.6651	0.073*	
H14B	0.0392	−0.2105	0.5785	0.073*	
C15	0.2126 (9)	−0.3179 (6)	0.6263 (4)	0.062 (2)	
H15A	0.1494	−0.3554	0.5825	0.075*	
H15B	0.1647	−0.3378	0.6702	0.075*	
C16	0.3524 (9)	−0.3477 (6)	0.6340 (4)	0.057 (2)	
H16A	0.4185	−0.3072	0.6758	0.068*	
H16B	0.3357	−0.4225	0.6429	0.068*	
C17	−0.3083 (9)	0.3740 (6)	0.1718 (4)	0.041 (2)	
C18	−0.4398 (9)	0.3943 (6)	0.1667 (4)	0.053 (2)	
H18	−0.5230	0.3411	0.1492	0.063*	
C19	−0.4496 (10)	0.4936 (8)	0.1874 (4)	0.062 (2)	
H19	−0.5392	0.5072	0.1850	0.074*	
C20	−0.3253 (11)	0.5724 (6)	0.2114 (4)	0.063 (3)	
H20	−0.3319	0.6394	0.2252	0.075*	
C21	−0.1925 (9)	0.5539 (6)	0.2154 (4)	0.055 (2)	
H21	−0.1096	0.6082	0.2316	0.066*	
C22	−0.1822 (8)	0.4550 (6)	0.1955 (4)	0.041 (2)	
C23	0.0801 (9)	0.5023 (7)	0.2165 (5)	0.073 (3)	
H23A	0.1502	0.4686	0.2405	0.087*	
H23B	0.0705	0.5561	0.2517	0.087*	
C24	0.1317 (9)	0.5524 (6)	0.1490 (5)	0.078 (3)	
H24A	0.0596	0.5825	0.1228	0.093*	
H24B	0.2197	0.6089	0.1629	0.093*	
C25	0.2142 (9)	0.5112 (6)	0.0356 (5)	0.072 (3)	
H25A	0.3008	0.5694	0.0479	0.086*	
H25B	0.1436	0.5367	0.0037	0.086*	
C26	0.2478 (9)	0.4232 (7)	−0.0036 (4)	0.069 (3)	
H26A	0.2912	0.4483	−0.0479	0.083*	
H26B	0.3167	0.3973	0.0292	0.083*	
C27	0.1488 (10)	0.2524 (6)	−0.0615 (5)	0.086 (3)	
H27A	0.2114	0.2218	−0.0279	0.104*	
H27B	0.1961	0.2747	−0.1053	0.104*	
C28	0.0099 (11)	0.1736 (6)	−0.0844 (5)	0.077 (3)	
H28A	−0.0567	0.2067	−0.1127	0.093*	
H28B	0.0234	0.1176	−0.1163	0.093*	
C29	−0.1779 (10)	0.0482 (6)	−0.0361 (5)	0.071 (3)	
H29A	−0.1581	−0.0139	−0.0576	0.085*	
H29B	−0.2448	0.0707	−0.0725	0.085*	
C30	−0.2434 (9)	0.0212 (6)	0.0333 (5)	0.075 (3)	
H30A	−0.3298	−0.0376	0.0232	0.089*	
H30B	−0.1759	0.0014	0.0710	0.089*	
C31	−0.3593 (9)	0.0944 (6)	0.1204 (5)	0.072 (3)	
H31A	−0.2996	0.0822	0.1649	0.087*	
H31B	−0.4414	0.0327	0.1088	0.087*	
C32	−0.4091 (9)	0.1910 (5)	0.1331 (5)	0.073 (3)	
H32A	−0.4644	0.2052	0.0875	0.088*	
H32B	−0.4704	0.1802	0.1726	0.088*	
C33	0.0053 (8)	−0.2538 (7)	0.3610 (4)	0.050 (2)	
C34	0.1016 (9)	0.1047 (7)	0.2239 (4)	0.055 (2)	
C35	−0.3980 (10)	−0.1725 (6)	0.1608 (5)	0.054 (2)	

### Atomic displacement parameters (Å^2^)

**Table:**

	*U*^11^	*U*^22^	*U*^33^	*U*^12^	*U*^13^	*U*^23^
Cu1	0.0681 (8)	0.0618 (7)	0.0576 (7)	0.0144 (6)	0.0103 (6)	−0.0051 (6)
K1	0.0542 (12)	0.0595 (12)	0.0424 (11)	0.0270 (10)	0.0131 (9)	0.0074 (9)
K2	0.0580 (13)	0.0523 (11)	0.0607 (13)	0.0137 (10)	0.0162 (10)	−0.0039 (10)
N1	0.070 (6)	0.076 (6)	0.067 (6)	0.023 (4)	−0.005 (4)	0.010 (4)
N2	0.069 (6)	0.059 (5)	0.064 (5)	0.005 (4)	0.013 (4)	−0.002 (4)
N3	0.063 (6)	0.067 (5)	0.062 (6)	0.014 (4)	0.009 (4)	0.000 (4)
O1	0.065 (4)	0.060 (3)	0.047 (4)	0.030 (3)	0.026 (3)	0.023 (3)
O2	0.061 (4)	0.063 (4)	0.035 (3)	0.032 (3)	0.016 (3)	0.005 (3)
O3	0.053 (4)	0.058 (4)	0.043 (3)	0.023 (3)	0.002 (3)	0.007 (3)
O4	0.041 (4)	0.060 (4)	0.052 (4)	0.021 (3)	0.000 (3)	0.006 (3)
O5	0.052 (4)	0.059 (4)	0.056 (4)	0.023 (3)	0.009 (3)	−0.005 (3)
O6	0.060 (4)	0.054 (4)	0.051 (4)	0.024 (3)	0.017 (3)	0.000 (3)
O7	0.050 (4)	0.034 (3)	0.072 (4)	0.006 (3)	0.018 (3)	−0.003 (3)
O8	0.047 (4)	0.062 (4)	0.064 (4)	0.008 (3)	0.003 (3)	−0.018 (3)
O9	0.075 (4)	0.059 (4)	0.054 (4)	0.007 (3)	0.026 (3)	0.000 (3)
O10	0.066 (4)	0.045 (3)	0.066 (4)	0.017 (3)	0.024 (3)	0.004 (3)
O11	0.079 (5)	0.054 (4)	0.060 (4)	0.022 (3)	0.019 (4)	0.006 (3)
O12	0.071 (4)	0.044 (3)	0.082 (4)	0.020 (3)	0.027 (4)	0.001 (3)
S1	0.0656 (17)	0.0853 (17)	0.0639 (17)	0.0266 (14)	0.0065 (13)	0.0060 (14)
S2	0.0675 (19)	0.0794 (18)	0.124 (2)	0.0000 (15)	0.0018 (17)	0.0385 (17)
S3	0.0670 (17)	0.0811 (18)	0.0795 (19)	−0.0015 (14)	−0.0045 (15)	0.0047 (14)
C1	0.041 (5)	0.042 (5)	0.048 (6)	0.020 (4)	−0.003 (5)	−0.007 (4)
C2	0.071 (7)	0.072 (6)	0.052 (6)	0.031 (5)	−0.011 (5)	0.000 (5)
C3	0.056 (7)	0.079 (7)	0.095 (9)	0.037 (6)	−0.018 (6)	0.001 (6)
C4	0.055 (7)	0.079 (7)	0.097 (9)	0.034 (6)	0.004 (7)	−0.007 (7)
C5	0.053 (6)	0.055 (6)	0.069 (7)	0.020 (5)	0.011 (5)	−0.003 (5)
C6	0.044 (6)	0.039 (5)	0.049 (6)	0.010 (4)	−0.001 (5)	0.001 (4)
C7	0.066 (6)	0.061 (6)	0.046 (6)	0.017 (5)	0.019 (5)	−0.012 (4)
C8	0.068 (7)	0.069 (6)	0.040 (5)	0.017 (5)	0.019 (5)	0.003 (5)
C9	0.049 (6)	0.090 (7)	0.036 (5)	0.027 (5)	−0.003 (4)	0.013 (5)
C10	0.052 (6)	0.071 (6)	0.069 (7)	0.030 (5)	−0.001 (5)	0.020 (5)
C11	0.042 (6)	0.064 (6)	0.068 (7)	0.022 (5)	−0.006 (5)	0.012 (5)
C12	0.073 (7)	0.055 (6)	0.065 (7)	0.035 (5)	−0.003 (5)	−0.009 (5)
C13	0.056 (6)	0.076 (7)	0.069 (7)	0.041 (5)	0.018 (5)	−0.004 (5)
C14	0.052 (6)	0.075 (6)	0.069 (6)	0.033 (5)	0.027 (5)	0.004 (5)
C15	0.081 (7)	0.067 (6)	0.047 (6)	0.023 (6)	0.032 (5)	0.021 (5)
C16	0.071 (7)	0.056 (5)	0.056 (6)	0.029 (5)	0.027 (5)	0.030 (4)
C17	0.045 (6)	0.055 (6)	0.027 (5)	0.015 (5)	0.015 (4)	0.013 (4)
C18	0.057 (6)	0.050 (6)	0.053 (6)	0.017 (5)	0.008 (5)	0.000 (4)
C19	0.066 (7)	0.091 (7)	0.048 (6)	0.045 (6)	0.028 (5)	0.010 (5)
C20	0.092 (8)	0.053 (6)	0.061 (6)	0.043 (6)	0.027 (6)	0.010 (5)
C21	0.070 (7)	0.055 (6)	0.040 (5)	0.015 (5)	0.014 (5)	−0.009 (4)
C22	0.041 (5)	0.050 (5)	0.038 (5)	0.019 (5)	0.012 (4)	0.002 (4)
C23	0.057 (7)	0.086 (7)	0.060 (7)	0.001 (5)	−0.001 (5)	−0.023 (5)
C24	0.075 (7)	0.058 (6)	0.082 (8)	−0.012 (5)	0.013 (6)	−0.021 (6)
C25	0.081 (7)	0.062 (6)	0.063 (7)	−0.004 (5)	0.026 (6)	0.005 (5)
C26	0.080 (8)	0.087 (7)	0.046 (6)	0.020 (6)	0.026 (5)	0.013 (5)
C27	0.120 (10)	0.059 (6)	0.099 (8)	0.033 (6)	0.069 (8)	0.010 (6)
C28	0.136 (10)	0.057 (6)	0.049 (6)	0.037 (6)	0.028 (7)	−0.003 (5)
C29	0.079 (7)	0.045 (6)	0.087 (8)	0.014 (5)	0.016 (6)	−0.016 (5)
C30	0.085 (7)	0.058 (6)	0.090 (8)	0.025 (5)	0.032 (6)	−0.004 (6)
C31	0.082 (7)	0.035 (5)	0.097 (8)	0.001 (5)	0.035 (6)	0.006 (5)
C32	0.062 (6)	0.035 (5)	0.117 (8)	−0.004 (5)	0.030 (6)	−0.008 (5)
C33	0.039 (5)	0.066 (6)	0.040 (5)	0.009 (5)	0.000 (4)	−0.012 (5)
C34	0.052 (6)	0.067 (6)	0.043 (6)	0.022 (5)	−0.014 (5)	−0.002 (5)
C35	0.062 (7)	0.045 (5)	0.055 (6)	0.014 (5)	0.015 (5)	0.002 (5)

### Geometric parameters (Å, °)

**Table:**

Cu1—N1	1.873 (8)	C7—H7A	0.970
Cu1—N2	1.910 (7)	C7—H7B	0.970
Cu1—N3	1.915 (7)	C8—H8A	0.970
K1—O5	2.683 (5)	C8—H8B	0.970
K1—O2	2.718 (5)	C9—C10	1.510 (9)
K1—O1	2.727 (5)	C9—H9A	0.970
K1—O3	2.756 (5)	C9—H9B	0.970
K1—O6	2.761 (5)	C10—H10A	0.970
K1—O4	2.875 (5)	C10—H10B	0.970
K1—O4^i^	2.903 (5)	C11—C12	1.489 (9)
K1—S1	3.317 (3)	C11—H11A	0.970
K2—O12	2.729 (5)	C11—H11B	0.970
K2—O11	2.730 (5)	C12—H12A	0.970
K2—O9	2.765 (5)	C12—H12B	0.970
K2—O8	2.773 (5)	C13—C14	1.479 (9)
K2—O10	2.797 (5)	C13—H13A	0.970
K2—O7	2.819 (5)	C13—H13B	0.970
K2—S2	3.198 (3)	C14—H14A	0.970
N1—C33	1.185 (9)	C14—H14B	0.970
N2—C34	1.155 (8)	C15—C16	1.501 (9)
N3—C35	1.129 (9)	C15—H15A	0.970
O1—C1	1.384 (8)	C15—H15B	0.970
O1—C16	1.421 (7)	C16—H16A	0.970
O2—C6	1.370 (8)	C16—H16B	0.970
O2—C7	1.443 (7)	C17—C18	1.364 (9)
O3—C8	1.426 (7)	C17—C22	1.402 (9)
O3—C9	1.435 (8)	C18—C19	1.379 (9)
O4—C11	1.419 (8)	C18—H18	0.930
O4—C10	1.437 (8)	C19—C20	1.376 (10)
O4—K1^i^	2.903 (5)	C19—H19	0.930
O5—C13	1.411 (8)	C20—C21	1.367 (10)
O5—C12	1.420 (8)	C20—H20	0.930
O6—C15	1.408 (7)	C21—C22	1.373 (9)
O6—C14	1.433 (8)	C21—H21	0.930
O7—C17	1.379 (8)	C23—C24	1.468 (10)
O7—C32	1.421 (8)	C23—H23A	0.970
O8—C22	1.376 (8)	C23—H23B	0.970
O8—C23	1.431 (8)	C24—H24A	0.970
O9—C25	1.411 (8)	C24—H24B	0.970
O9—C24	1.427 (8)	C25—C26	1.470 (9)
O10—C26	1.392 (8)	C25—H25A	0.970
O10—C27	1.425 (8)	C25—H25B	0.970
O11—C28	1.400 (8)	C26—H26A	0.970
O11—C29	1.422 (8)	C26—H26B	0.970
O12—C31	1.423 (8)	C27—C28	1.468 (11)
O12—C30	1.425 (7)	C27—H27A	0.970
S1—C33	1.626 (9)	C27—H27B	0.970
S2—C34	1.622 (9)	C28—H28A	0.970
S3—C35	1.625 (9)	C28—H28B	0.970
C1—C2	1.359 (10)	C29—C30	1.476 (10)
C1—C6	1.388 (9)	C29—H29A	0.970
C2—C3	1.377 (10)	C29—H29B	0.970
C2—H2	0.930	C30—H30A	0.970
C3—C4	1.372 (11)	C30—H30B	0.970
C3—H3	0.930	C31—C32	1.495 (9)
C4—C5	1.369 (10)	C31—H31A	0.970
C4—H4	0.930	C31—H31B	0.970
C5—C6	1.361 (9)	C32—H32A	0.970
C5—H5	0.930	C32—H32B	0.970
C7—C8	1.483 (9)		
			
N1—Cu1—N2	121.2 (3)	C10—C9—H9A	110.3
N1—Cu1—N3	121.5 (3)	O3—C9—H9B	110.3
N2—Cu1—N3	117.1 (3)	C10—C9—H9B	110.3
O5—K1—O2	170.74 (16)	H9A—C9—H9B	108.6
O5—K1—O1	123.77 (17)	O4—C10—C9	111.5 (6)
O2—K1—O1	56.17 (15)	O4—C10—H10A	109.3
O5—K1—O3	117.82 (17)	C9—C10—H10A	109.3
O2—K1—O3	61.31 (15)	O4—C10—H10B	109.3
O1—K1—O3	117.47 (16)	C9—C10—H10B	109.3
O5—K1—O6	62.87 (16)	H10A—C10—H10B	108.0
O2—K1—O6	116.73 (16)	O4—C11—C12	109.8 (6)
O1—K1—O6	60.98 (15)	O4—C11—H11A	109.7
O3—K1—O6	172.87 (16)	C12—C11—H11A	109.7
O5—K1—O4	62.10 (16)	O4—C11—H11B	109.7
O2—K1—O4	120.45 (16)	C12—C11—H11B	109.7
O1—K1—O4	165.95 (16)	H11A—C11—H11B	108.2
O3—K1—O4	61.15 (15)	O5—C12—C11	109.1 (6)
O6—K1—O4	122.24 (16)	O5—C12—H12A	109.9
O5—K1—O4^i^	97.66 (15)	C11—C12—H12A	109.9
O2—K1—O4^i^	91.54 (15)	O5—C12—H12B	109.9
O1—K1—O4^i^	83.25 (14)	C11—C12—H12B	109.9
O3—K1—O4^i^	98.96 (15)	H12A—C12—H12B	108.3
O6—K1—O4^i^	87.85 (15)	O5—C13—C14	109.4 (6)
O4—K1—O4^i^	83.24 (15)	O5—C13—H13A	109.8
O5—K1—S1	88.66 (12)	C14—C13—H13A	109.8
O2—K1—S1	82.11 (12)	O5—C13—H13B	109.8
O1—K1—S1	83.85 (12)	C14—C13—H13B	109.8
O3—K1—S1	87.91 (12)	H13A—C13—H13B	108.2
O6—K1—S1	85.01 (12)	O6—C14—C13	109.0 (6)
O4—K1—S1	109.70 (12)	O6—C14—H14A	109.9
O4^i^—K1—S1	167.06 (11)	C13—C14—H14A	109.9
O12—K2—O11	60.81 (17)	O6—C14—H14B	109.9
O12—K2—O9	158.87 (17)	C13—C14—H14B	109.9
O11—K2—O9	120.39 (18)	H14A—C14—H14B	108.3
O12—K2—O8	113.47 (17)	O6—C15—C16	109.4 (6)
O11—K2—O8	169.50 (18)	O6—C15—H15A	109.8
O9—K2—O8	61.31 (15)	C16—C15—H15A	109.8
O12—K2—O10	114.86 (17)	O6—C15—H15B	109.8
O11—K2—O10	61.28 (16)	C16—C15—H15B	109.8
O9—K2—O10	59.47 (15)	H15A—C15—H15B	108.2
O8—K2—O10	118.10 (16)	O1—C16—C15	107.2 (6)
O12—K2—O7	59.33 (15)	O1—C16—H16A	110.3
O11—K2—O7	118.88 (17)	C15—C16—H16A	110.3
O9—K2—O7	111.00 (15)	O1—C16—H16B	110.3
O8—K2—O7	54.23 (15)	C15—C16—H16B	110.3
O10—K2—O7	141.73 (16)	H16A—C16—H16B	108.5
O12—K2—S2	115.35 (13)	C18—C17—O7	125.4 (7)
O11—K2—S2	97.40 (13)	C18—C17—C22	120.1 (7)
O9—K2—S2	85.75 (13)	O7—C17—C22	114.5 (7)
O8—K2—S2	93.05 (14)	C17—C18—C19	120.2 (8)
O10—K2—S2	99.01 (13)	C17—C18—H18	119.9
O7—K2—S2	117.94 (13)	C19—C18—H18	119.9
C33—N1—Cu1	168.9 (7)	C20—C19—C18	119.3 (8)
C34—N2—Cu1	176.3 (7)	C20—C19—H19	120.3
C35—N3—Cu1	168.5 (8)	C18—C19—H19	120.3
C1—O1—C16	117.6 (6)	C21—C20—C19	121.3 (8)
C1—O1—K1	119.3 (4)	C21—C20—H20	119.4
C16—O1—K1	118.6 (4)	C19—C20—H20	119.4
C6—O2—C7	118.6 (6)	C20—C21—C22	119.7 (8)
C6—O2—K1	119.9 (4)	C20—C21—H21	120.2
C7—O2—K1	117.4 (4)	C22—C21—H21	120.2
C8—O3—C9	113.0 (6)	C21—C22—O8	125.8 (7)
C8—O3—K1	102.9 (4)	C21—C22—C17	119.5 (7)
C9—O3—K1	106.0 (4)	O8—C22—C17	114.7 (7)
C11—O4—C10	113.2 (6)	O8—C23—C24	111.1 (7)
C11—O4—K1	106.6 (4)	O8—C23—H23A	109.4
C10—O4—K1	112.5 (4)	C24—C23—H23A	109.4
C11—O4—K1^i^	120.6 (4)	O8—C23—H23B	109.4
C10—O4—K1^i^	106.1 (4)	C24—C23—H23B	109.4
K1—O4—K1^i^	96.76 (15)	H23A—C23—H23B	108.0
C13—O5—C12	113.7 (6)	O9—C24—C23	108.1 (7)
C13—O5—K1	114.9 (4)	O9—C24—H24A	110.1
C12—O5—K1	116.6 (4)	C23—C24—H24A	110.1
C15—O6—C14	113.6 (6)	O9—C24—H24B	110.1
C15—O6—K1	108.0 (4)	C23—C24—H24B	110.1
C14—O6—K1	109.3 (4)	H24A—C24—H24B	108.4
C17—O7—C32	117.2 (6)	O9—C25—C26	107.9 (7)
C17—O7—K2	119.5 (4)	O9—C25—H25A	110.1
C32—O7—K2	117.2 (4)	C26—C25—H25A	110.1
C22—O8—C23	119.9 (6)	O9—C25—H25B	110.1
C22—O8—K2	120.9 (4)	C26—C25—H25B	110.1
C23—O8—K2	109.5 (4)	H25A—C25—H25B	108.4
C25—O9—C24	114.4 (6)	O10—C26—C25	110.6 (7)
C25—O9—K2	118.5 (4)	O10—C26—H26A	109.5
C24—O9—K2	116.9 (4)	C25—C26—H26A	109.5
C26—O10—C27	113.3 (6)	O10—C26—H26B	109.5
C26—O10—K2	113.1 (4)	C25—C26—H26B	109.5
C27—O10—K2	109.5 (4)	H26A—C26—H26B	108.1
C28—O11—C29	114.1 (7)	O10—C27—C28	108.6 (7)
C28—O11—K2	115.8 (4)	O10—C27—H27A	110.0
C29—O11—K2	116.8 (4)	C28—C27—H27A	110.0
C31—O12—C30	112.4 (6)	O10—C27—H27B	110.0
C31—O12—K2	110.9 (4)	C28—C27—H27B	110.0
C30—O12—K2	109.4 (4)	H27A—C27—H27B	108.3
C33—S1—K1	93.0 (3)	O11—C28—C27	109.4 (8)
C34—S2—K2	89.6 (3)	O11—C28—H28A	109.8
C2—C1—O1	125.6 (8)	C27—C28—H28A	109.8
C2—C1—C6	119.3 (8)	O11—C28—H28B	109.8
O1—C1—C6	115.1 (7)	C27—C28—H28B	109.8
C1—C2—C3	120.6 (9)	H28A—C28—H28B	108.3
C1—C2—H2	119.7	O11—C29—C30	110.3 (7)
C3—C2—H2	119.7	O11—C29—H29A	109.6
C4—C3—C2	120.1 (9)	C30—C29—H29A	109.6
C4—C3—H3	120.0	O11—C29—H29B	109.6
C2—C3—H3	120.0	C30—C29—H29B	109.6
C5—C4—C3	119.2 (9)	H29A—C29—H29B	108.1
C5—C4—H4	120.4	O12—C30—C29	106.5 (7)
C3—C4—H4	120.4	O12—C30—H30A	110.4
C6—C5—C4	121.0 (9)	C29—C30—H30A	110.4
C6—C5—H5	119.5	O12—C30—H30B	110.4
C4—C5—H5	119.5	C29—C30—H30B	110.4
C5—C6—O2	124.8 (8)	H30A—C30—H30B	108.6
C5—C6—C1	119.8 (8)	O12—C31—C32	106.8 (6)
O2—C6—C1	115.4 (7)	O12—C31—H31A	110.4
O2—C7—C8	107.3 (6)	C32—C31—H31A	110.4
O2—C7—H7A	110.2	O12—C31—H31B	110.4
C8—C7—H7A	110.2	C32—C31—H31B	110.4
O2—C7—H7B	110.2	H31A—C31—H31B	108.6
C8—C7—H7B	110.2	O7—C32—C31	108.1 (7)
H7A—C7—H7B	108.5	O7—C32—H32A	110.1
O3—C8—C7	108.7 (7)	C31—C32—H32A	110.1
O3—C8—H8A	110.0	O7—C32—H32B	110.1
C7—C8—H8A	110.0	C31—C32—H32B	110.1
O3—C8—H8B	110.0	H32A—C32—H32B	108.4
C7—C8—H8B	110.0	N1—C33—S1	177.6 (8)
H8A—C8—H8B	108.3	N2—C34—S2	179.7 (10)
O3—C9—C10	107.0 (6)	N3—C35—S3	179.0 (9)
O3—C9—H9A	110.3		

Symmetry codes: (i) −*x*+1, −*y*, −*z*+1.
